# Effect of Clutter Filter in High-Frame-Rate Ultrasonic Backscatter Coefficient Analysis

**DOI:** 10.3390/s23052639

**Published:** 2023-02-27

**Authors:** Masaaki Omura, Kunimasa Yagi, Ryo Nagaoka, Kenji Yoshida, Tadashi Yamaguchi, Hideyuki Hasegawa

**Affiliations:** 1 Faculty of Engineering, University of Toyama, Toyama 930-8555, Japan; 2 School of Medicine, Kanazawa Medical University, Kanazawa 920-0293, Japan; 3 Center for Frontier Medical Engineering, Chiba University, Chiba 263-8522, Japan

**Keywords:** quantitative ultrasound, high-frame-rate imaging, backscatter coefficient, clutter filter, hemorheology, red blood cell

## Abstract

High-frame-rate imaging with a clutter filter can clearly visualize blood flow signals and provide more efficient discrimination with tissue signals. In vitro studies using clutter-less phantom and high-frequency ultrasound suggested a possibility of evaluating the red blood cell (RBC) aggregation by analyzing the frequency dependence of the backscatter coefficient (*BSC*). However, in in vivo applications, clutter filtering is required to visualize echoes from the RBC. This study initially evaluated the effect of the clutter filter for ultrasonic *BSC* analysis for in vitro and preliminary in vivo data to characterize hemorheology. Coherently compounded plane wave imaging at a frame rate of 2 kHz was carried out in high-frame-rate imaging. Two samples of RBCs suspended by saline and autologous plasma for in vitro data were circulated in two types of flow phantoms without or with clutter signals. The singular value decomposition was applied to suppress the clutter signal in the flow phantom. The *BSC* was calculated using the reference phantom method, and it was parametrized by spectral slope and mid-band fit (MBF) between 4–12 MHz. The velocity distribution was estimated by the block matching method, and the shear rate was estimated by the least squares approximation of the slope near the wall. Consequently, the spectral slope of the saline sample was always around four (Rayleigh scattering), independently of the shear rate, because the RBCs did not aggregate in the solution. Conversely, the spectral slope of the plasma sample was lower than four at low shear rates but approached four by increasing the shear rate, because the aggregations were presumably dissolved by the high shear rate. Moreover, the MBF of the plasma sample decreased from −36 to −49 dB in both flow phantoms with increasing shear rates, from approximately 10 to 100 s^−1^. The variation in the spectral slope and MBF in the saline sample was comparable to the results of in vivo cases in healthy human jugular veins when the tissue and blood flow signals could be separated.

## 1. Introduction

The viscoelasticity of blood has been noted as a hemodynamic factor for impaired vascular function [1]. The flow field of blood (velocity gradient, i.e., shear rate) is affected by the hemodynamic property of blood [2,3]. The most dominant component of blood is the red blood cell (RBC), and the flow pattern depends on its behavior. The backscattered signal from the RBC is related to the biochemical properties of the blood sample, e.g., the hematocrit and the level of aggregation. The interaction of the RBC with plasma proteins results in the formation of RBC aggregates, which increase the flow resistance and, thus, increase the viscosity of the blood at a low shear rate. Hence, the relationship between the physical properties and flow patterns of RBCs must be comprehended. The viscoelasticity of blood has been noted as a hemodynamic factor for impaired vascular function [1]. The flow field of blood (velocity gradient, i.e., shear rate) is affected by the hemodynamic property of blood [2,3]. The most dominant component of blood is the red blood cell (RBC), and the flow pattern depends on its behavior. The backscattered signal from the RBC is related to the biochemical properties of the blood sample, e.g., the hematocrit and the level of aggregation. The interaction of the RBC with plasma proteins results in the formation of RBC aggregates, which increase the flow resistance and, thus, increase the viscosity of the blood at a low shear rate. Hence, the relationship between the physical properties and flow patterns of RBCs must be comprehended.

High-frame-rate imaging with a clutter filter can clearly visualize blood flow signals and provide more efficient discrimination with tissue signals. The tissue signals are commonly removed using a high-pass finite or infinite impulse response filters [4]. The idea behind the clutter filter is that the low and high temporal frequency components are echoes from slowly moving tissues and blood flow signals, respectively. Singular value decomposition (SVD) is one of the advanced techniques for the clutter filter design [5,6,7,8]. An SVD-based clutter filter improves the extraction of tiny vessels with slow velocities, in addition to the sensitivity in clutter rejection between slow tissues and blood flow signals [9,10].

The degree of the RBC aggregation can be evaluated in high-frequency ultrasound by analyzing the frequency dependence of the ultrasonic backscatter signals, i.e., backscatter coefficient (*BSC*) [11,12,13,14]. The attenuation coefficient of blood has been measured through preliminary in vivo study in the frequency range of 10–45 MHz [15]. A pilot clinical study of quantitative *BSC* analysis has been demonstrated for the RBC aggregation measurement [16]. Conventionally focused line-by-line imaging with frame rates of several 10 s to 100 Hz is enough to observe moving RBCs at a low shear rate, and these studies have mainly focused on the hemorheology under peripheral circulation. An in vitro study has revealed the efficiency of high-frame-rate plane wave imaging with a 7.5-MHz ultrasound to characterize static tissue [17] and flowing RBCs in the agar phantom, i.e., in the clutter-less case [18]. Our previous study has also analyzed spatial features such as the contrast of flowing blood in in vitro experiments and in in vivo measurements of human jugular veins [19]. For in vivo imaging in the central vasomotor, such as a carotid artery and jugular vein, the clutter filter is required to visualize echoes from RBCs by suppressing tissue signals. However, the clutter filter has not been considered in the *BSC* analysis so far.

The novelty of this study is to develop non-invasive hemorheological imaging based on the *BSC* analysis for in vivo situations. In this study, the performance of the clutter filter was experimentally evaluated to indicate the hemorheological property dependently on a shear rate under physiological conditions. The spectral *BSC* analysis of porcine blood in a flow phantom without or with clutter signals was analyzed from low to high steady shear rates that were comparable with the human jugular vein [20]. Secondly, in vivo experiments in jugular veins of healthy subjects were carried out to confirm the feasibility under physiological flow conditions. The reproducibility and feasibility of high-frame-rate spectral analysis with clutter filters were compared among inter-subject variance.

## 2. Materials and Methods

### 2.1. In Vitro Porcine Blood Measurement

**Figure 1:**
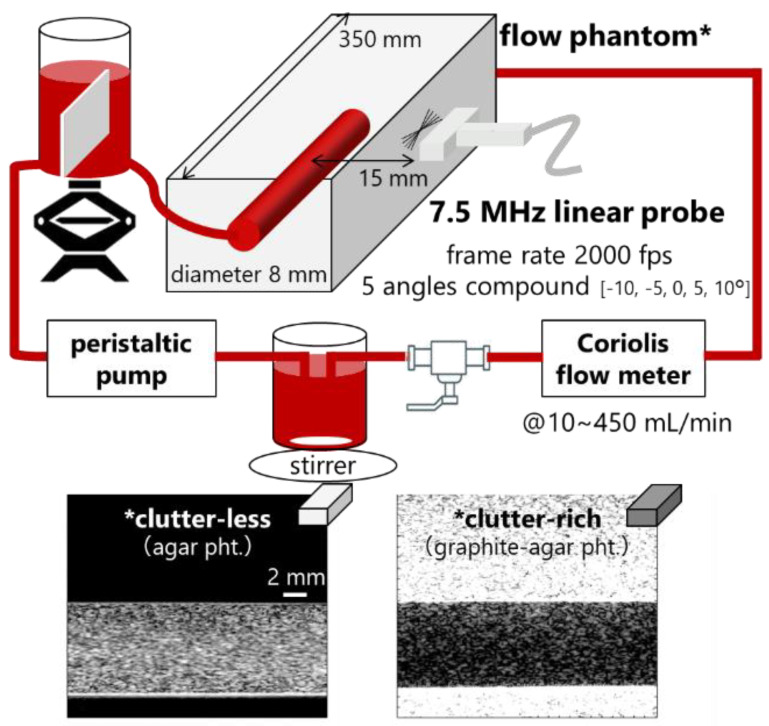
Illustration of in vitro measurement. Two flow phantoms (*) were compared in this study.

Whole porcine blood (within 24 h of its collection) anticoagulated by sodium citrate was centrifuged and separated into RBCs, plasma, and platelet at room temperature (24 °C), as followed in previous studies [18,21,22]. The RBCs were diluted in phosphate-buffered solution (PBS) or autologous plasma (40% hematocrit) to reproduce dispersed or aggregated RBCs. Each sample was circulated at a steady flow in the cylindrical lumen surrounded by 2% agar (i.e., clutter-less) or 2% agar-7% graphite (i.e., clutter-rich) flow phantoms as illustrated in Figure 1. Acoustic properties of clutter-less and clutter-rich phantoms were as follows: speed of sound (SoS)—1500 and 1520 m/s; attenuation coefficient—0.05 and 0.52 dB/cm/MHz. The blood of 0.8 L was passed through the wall-less lumen (8 mm diameter and 350 mm length) in the flow phantom using a peristaltic pump (Model 07528-10, Masterflex). The distance from the phantom surface to the center lumen was 15 mm. The steady flow was controlled with a flow regulator (CY-1208, ASOH) and monitored with a Coriolis flowmeter (FD-SF8, Keyence). For in vitro experiment, ultrasound data were obtained at selected flow rates from 10 to 600 mL/min.

### 2.2. In Vivo Jugular Vein Measurement

All procedures were carried out following the ethical standards of the responsible committee on human experimentation and with the Helsinki Declaration of 1964 and later versions. The Ethics Committee of Toyama University Hospital approved the study protocol (IRB# R2015150 and R2019135). Informed consent was acquired from all subjects. The jugular veins of healthy subjects (n = 5, 21–49 y.o., male) were scanned along the long axis in vivo. All subjects were in the supine position and breathed normally during the measurement.

### 2.3. Data Acquisition and Post-Processing

A 7.5-MHz linear array probe (UST-5412, Fujifilm) was placed over the middle of the flow phantom so that the lumen along the long axis of the human jugular vein reached its maximum. Multiple plane waves at 5 angles of −10, −5, 0, 5, and 10 degrees were transmitted in each frame at pulse repetition periods of 96 μs, i.e., frame rate of about 2 kHz for compounded images. Tukey apodization with a coefficient of 0.4 was used in transmission. The radio frequency (RF) channel data were acquired at a sampling frequency of 31.25 MHz for 0.1 s (porcine blood experiment) and 0.96 s (in vivo measurement) using a research platform scanner (RSYS0016, Microsonic). The delay-and-sum method with the dynamic aperture (F-number of 1) was applied to the RF channel data [23]. The beamformed RF data (axial 24 mm × lateral 24 mm, 893 × 242 pixels) was reconstructed on a pixel-by-pixel basis (axial 25 μm × lateral 100 μm) at an SoS of 1540 m/s. The coherently compounded RF data were used for the following analysis.

### 2.4. Clutter Filter

**Figure 2:**
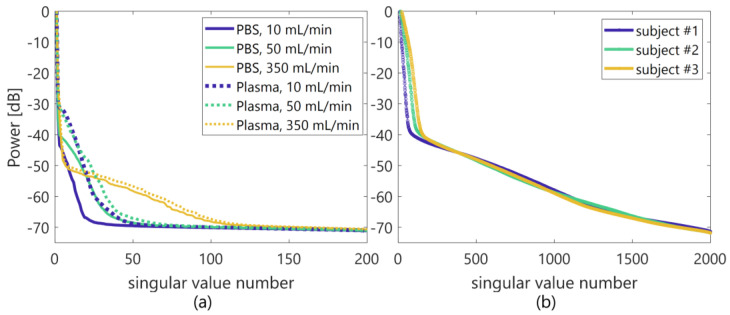
Illustration of the power curve of singular values (**a**,(**b**) porcine blood and in vivo jugular vein experiments.

A spatiotemporal filter based on SVD was applied to the beamformed RF data to emphasize the blood flow signal [9]. Briefly, a spatiotemporal matrix (spatial 216,106 pixels × temporal 200 (porcine blood experiment) or 2000 (in vivo measurement) frames) composed of the coherently compounded beamformed signals in all frames was used for the SVD filter. The spatiotemporal matrix S is decomposed by SVD into a product of three matrices as

(1) $$S=\hat{U}\hat{\Sigma }{\hat{V}}^{T},$$

where $\hat{U}$ and $\hat{V}$ are matrices in terms of spatial and temporal singular vectors, respectively, $\hat{\Sigma }$ is a diagonal matrix of singular values with descending order, and ^T^ is the transpose operation. The clutter-filtered signal $S^{\prime }$ is defined as

(2) $$S^{\prime }=\hat{U}\hat{\Sigma^{\prime }}{\hat{V}}^{T},$$

where $\hat{\Sigma^{\prime }}$ is a diagonal matrix in which the components in the matrix of $\hat{\Sigma }$ at orders less than the lower threshold and those higher than the higher threshold are replaced with zeros. The low- and high-rank thresholds of singular values for clutter, blood flow, and system noise components were empirically set among the porcine blood experiments (from −67 to −27 dB normalized by the maximum singular value from a reference phantom in Section 2.5) and in vivo (from −67 to −37 dB similar to the normalization criteria in the porcine experiments) measurements, respectively, in reference to the power curve of singular values, as shown in Figure 2. The 1st inflection point was assumed to be the boundary between clutter and blood flow signals, and 2nd inflection point was the boundary between blood flow signal and system noise.

### 2.5. Backscatter Coefficient Analysis

The *BSC* of the RBCs was calculated using the reference phantom method [24,25,26] in the frequency domain expressed as

(3) $$BSC\left(f,d\right)=\frac{\bar{P\left(f,d\right)}}{\bar{P_{ref}\left(f,d\right)}}BSC_{ref}\left(f\right)\exp\left[\frac{4df\left(\alpha -\alpha_{ref}\right)}{8.686}\right],$$

where $\bar{P}$ and $\bar{P_{ref}}$ are the mean of power spectra obtained from the RBCs and a reference medium, respectively; $BSC_{ref}$ is the theoretical *BSC*, d is the distance between the transducer and central position of the analysis window; and α and $\alpha_{ref}$ are the attenuation coefficients of the RBCs and the reference medium, respectively. The window size for calculation of the mean of the power spectra was 128 × 30 pixels (3.2 × 3.0 mm^2^, with 10 wavelengths in axial × 5-point spread functions in lateral direction under the SoS of 1540 m/s and center frequency of 7.5 MHz), and then ensemble averaging was performed for 4 frames (1.92 ms). Adjacent analysis windows overlapped by 80% in both the axial and lateral directions.

For in vitro measurement data, the reference medium consisted of a suspension of the RBC in saline at a low hematocrit [27]. In this study, a PBS sample at a hematocrit of 3% flowing in the geometry, as in Figure 1, in the clutter-less and clutter-rich phantoms at a flow rate of 10 mL/min, was used for the reference medium. The reference phantom used for in vivo analysis was made from 96.6 wt% purified water, 2 wt% agar, 0.9 wt% dispersant, and 0.5 wt% polyamide particles with a diameter of 10 μm (Orgasol 2002 EXD NAT1, Arkema). The means of SoS and attenuation coefficient of the reference phantom were 1503 m/s and 0.10 dB/cm/MHz, respectively, in the reflector method [17,28] using a single element plane transducer (V312, Olympus), as well as the backscatter power comparable to human blood [29].

In the attenuation compensation function, the constants α and $\alpha_{ref}$ were obtained using a typical attenuation coefficient: 0.30 dB/MHz for in vitro study determined by the reflector measurement in addition to considering the literature value [30]; 0.50 dB/cm/MHz in the tissue [31]; 0.15 dB/cm/MHz in the blood [32] for in vivo study. $BSC_{ref}$ for in vitro measurement was the theoretical backscatter of human blood as defined by 5 × $10^{-31}$ $f^{4}\;m^{-1}\;{\mathrm{sr}}^{-1}$ [33]. $BSC_{ref}$ for in vivo reference phantom was calculated based on the Faran model [34]. Here, the $BSC_{ref}$ was calculated using the known parameters of the Faran model: particle diameter = 10 μm; volume fractions = 0.5%; SoS in particle’s material = 2300 m/s; SoS in the surrounding medium = 1500 m/s; particle density = 1.03 g/cm^3^; medium density = 1.0 g/cm^3^; and Poisson ratio = 0.42.

The frequency dependence of the *BSC* was also evaluated by fitting a function to the calculated *BSC* in the frequency range from 4 to 12 MHz. The fitting function is expressed as

(4) $$10\log_{10}\left[BSC\left(f,d\right)\right]\approx n\log_{10}f+10\log_{10}b$$

Equation (4) could be modeled by a line (y=nx+B) having spectral slope n and intercept B in the least-square method, where B=$10\log_{10}b$. It means that the curve fitting was carried out in log scale. Also, the ideal estimate of n of the RBC has been expected to be 4 dB/MHz. In addition, the mid-band fit (MBF) was computed as the magnitude of the *BSC*, i.e., $10\log_{10}\left[BSC\left(7.5\;\mathrm{MHz},d\right)\right]$, at the frequency of 7.5 MHz.

### 2.6. Shear Rate Estimation

A block matching analysis was carried out to calculate the blood flow velocity. The input of the block matching analysis was the amplitude envelope of the coherently compounded beamformed signal. The block size was 60 × 60 pixels (1.4 × 6.0 mm^2^ for the axial and lateral directions), and the search distance in both directions was 40 pixels in reference to our previous numerical and experimental studies [29,35]. Adjacent macro blocks overlapped each other by 0.5 and 1.0 mm in the axial and lateral directions, respectively. The normalized cross-correlation function was up-sampled using the reconstructive interpolation method to estimate a sub-sample displacement [36,37]. Both the lateral and axial interpolation factors were 4. Also, the ensemble average of the correlation function was performed for 1.92 ms (4 frames).

The slope of flow velocity profile along the axial direction was calculated as the shear rate in the neighborhood around the proximal and distal boundaries between vein wall and lumen, which were manually traced. The gate length was 3 mm from each boundary in each beam line to estimate the slope by the least-squares method.

## 3. Results

**Figure 3:**
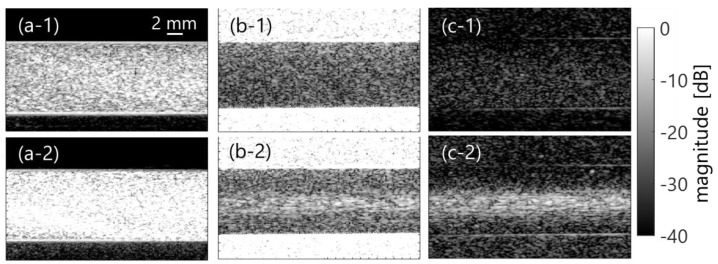
B-mode images in the porcine blood experiment in case of low shear rate. (**a**) Clutter-less case. (**b**,**c**) Clutter-rich cases without and with filters. (**1**) and (**2**) PBS and plasma samples.

**Figure 4:**
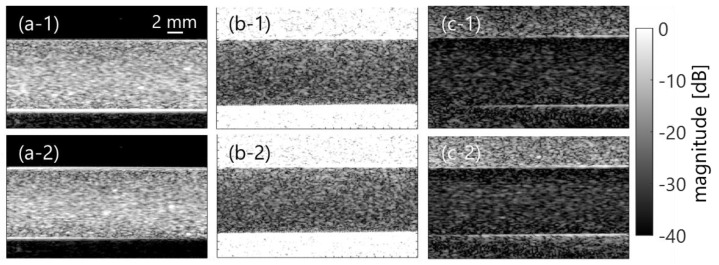
B-mode images in the porcine blood experiment in case of high shear rate, as well as Figure 3.

Figure 3a–c show examples of coherently compounded B-mode images in the clutter-less phantom without the clutter filter and clutter-rich phantoms without or with filters in the case of low flow rate (50 mL/min). Ensemble averaging was applied among 4 frames. Note that mean velocity and shear rate were from 3 to 4 cm/s and from 10 to 20 s^−1^ at the minimum to maximum, respectively. In addition, Figure 3(1)–(2) compare PBS and plasma samples, respectively. The echogenicity of the plasma sample was higher; however, the PBS sample had the same echogenicity that was independent of the shear rate. Conversely, B-mode images in the case of high flow rate (450 mL/min, mean velocity of 12–13 cm/s, and shear rate of 90–100 s^−1^) were equivalent when compared between PBS and plasma samples, as is shown in Figure 4.

**Figure 5:**
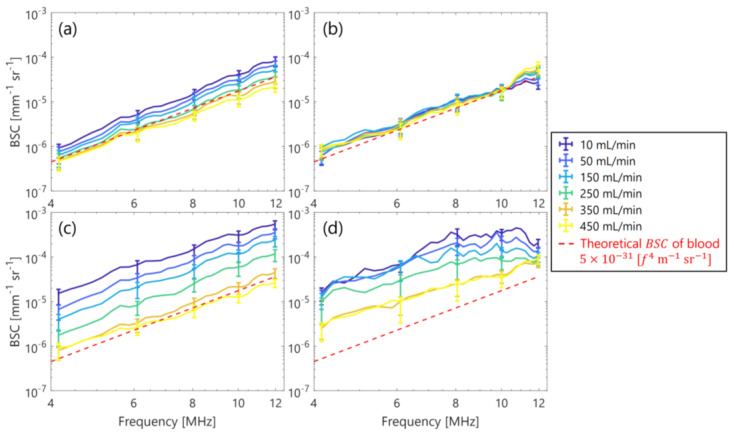
Frequency dependence of the *BSC* at selected flow rates. (**a**,**b**) PBS and plasma samples in the clutter-less phantom. (**c**,**d**) Those in the clutter-rich phantom.

**Figure 6:**
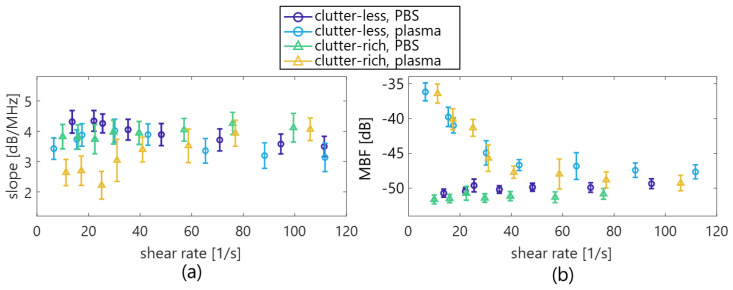
Shear rate dependence of the spectral slope (**a**) and MBF (**b**).

Figure 5 shows the typical *BSC* of the PBS and plasma samples in the clutter-less and clutter-rich phantoms at selected flow rates of 10, 50, 150, 250, 350, and 450 mL/min. The standard deviation is unmarked for clarity. The magnitude of the *BSC* in the plasma sample increased with a lower shear rate in both phantoms. In contrast, the magnitude of the *BSC* in the PBS sample was constant around the theoretical *BSC* of human blood [33], which was irrelevant to the flow rate. Additionally, as is seen in Figure 6a, the spectral slope of the PBS sample was always around 4 dB/MHz, i.e., Rayleigh scattering was independent of the shear rate, because the RBCs did not aggregate in the PBS solution. On the other hand, the spectral slope of the plasma sample, especially in the clutter-rich phantoms, was lower than four at low shear rates (2.2 dB/MHz at 25 s^−1^) but approached four by increasing the shear rate, because the aggregations were presumably dissolved by the high shear rate. Also, the MBF of the plasma sample decreased from −36 to −49 dB with an increasing shear rate, as is seen in Figure 6b. Conversely, the MBF of the PBS sample was constant around −52 to −49 dB and was unrelated to the shear rate.

**Figure 7:**
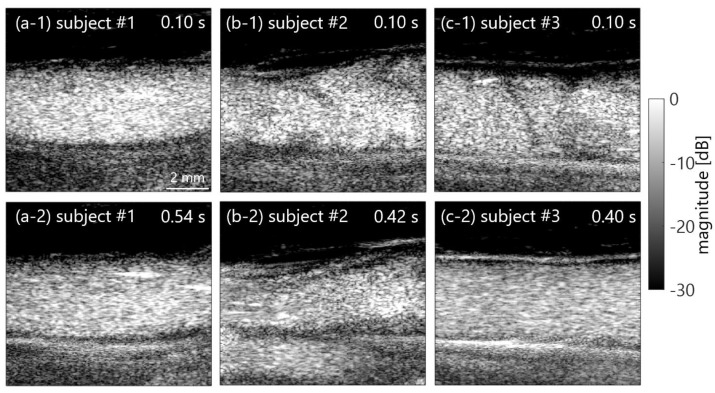
In vivo B-mode images in the human jugular vein in subjects #1 (**a**) to #3 (**c**) compared between low shear (**1**) and high shear rates (**2**) within the intra-subject.

**Figure 8:**
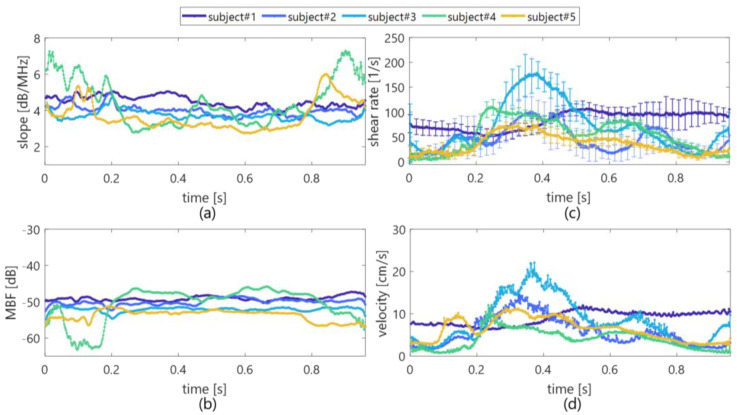
Temporal variation in the spectral slope (**a**), MBF (**b**), shear rate (**c**), and mean velocity (**d**) in each subject.

Typical B-mode images of in vivo jugular veins at selected low and high shear rates within intra-subjects are displayed in Figure 7a,b. Ensemble averaging was also applied among four frames to emphasize the signal-to-noise ratio. B-mode images were observed as the horizontal blurring effect of the diffusive suspensions; however, the echogenicity of blood flow could be clearly confirmed. Temporal variations in the spectral slope, MBF, shear rate (mean and standard deviation), and mean velocity are illustrated in Figure 8. Standard deviations in the *BSC* features and velocity are unmarked for clarity. For five healthy subjects, the spectral slope and MBF in Figure 8a,b were distributed around 2.8 to 5.0 dB/MHz and −53 to −46 dB within the approximate accelerating phase of the shear rate (e.g., increasing from less than 50 s^−1^ to over 100 s^−1^) through 0.2 to 0.4 s and the hereafter phase until around 0.8 s. In contrast, the spectral slope rapidly increased to over 5.0 dB/MHz at around 0.1 and 0.9 s (constant phase of the low shear rate and velocity) and exhibited a low MBF of less than −55 dB.

## 4. Discussion

This study initially evaluated the effect of a clutter filter in high-frame-rate ultrasonic backscatter coefficient analysis for blood characterization. In the porcine blood experiment, the property of the RBC aggregation was visualized in a low shear rate (around < 50 s^−1^) by comparing PBS and plasma samples, as is shown in Figure 3. The RBC aggregation by force between the cell and protein in the overall plasma would induce increasing echogenicity in accordance with previous studies [18,21,38,39]. In contrast, the RBC disaggregation that was caused by the high shear was confirmed through the lower echogenicity of both samples in Figure 4. These findings in the clutter-rich phantom were consistent with the previous studies on the dependence of ultrasonic backscatter power on the shear rate and hemorheology in the case of a clutter-less geometry [18,21,38,39]. In addition, the *BSC* features, such as spectral slope and MBF, could be quantitatively evaluated in the different shear rates, as is shown in Figure 6.

**Figure 9:**
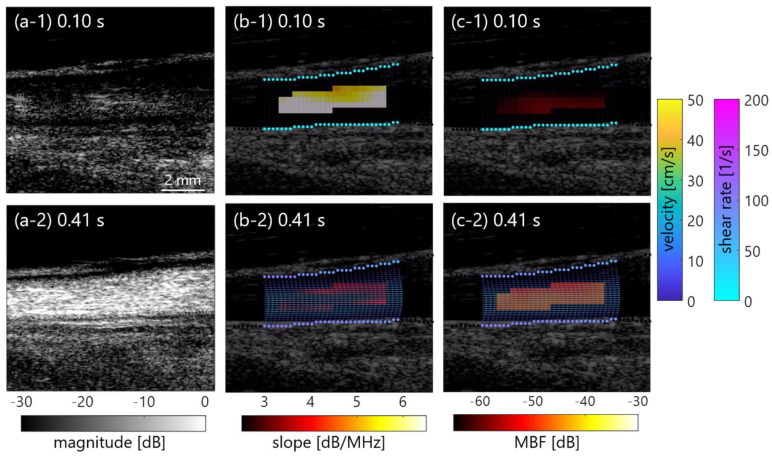
B-mode image (**a**) and spatial distribution of the spectral slope (**b**) and MBF (**c**) with velocity vector and shear rate in subject #4.

For five in vivo subjects within the accelerating and deaccelerating phases of shear rate, such as from 0.2 to 0.8 s, the spectral slope and MBF were comparable to those in in vitro experiments of PBS sample, and low shear plasma sample as can be seen in Figure 6 and Figure 8. The variability in *BSC* features among inter-subjects would be induced by the assumption of same attenuation and by the level of clutter rejection. Further investigation will be necessary to conduct an extensive clinical study to clarify the findings more explicitly in this preliminary study. Although the spectral slope and MBF in 3 of 5 subjects (#1 to #3) were also steady temporally, those in 2 of 5 subjects (#4 and #5) were rapidly changed in the constant phase of shear rate, such as around 0.1 and 0.9 s. Figure 9 displays typical B-mode images and spatial distributions of the spectral slope and MBF with vector velocity and shear rate. The echogenicity inside the vein was lower in Figure 9a, because the blood signals were suppressed by the clutter filter due to the near velocity or echogenicity between tissue and blood flow.

We further investigated the relationship of frequencies (in the clutter filter and *BSC* feature) between temporal and axial directions. The main temporal frequency was calculated by the column vectors ${\hat{v}}_{n}$ in the temporal singular matrix $\hat{V}=\left[{\hat{v}}_{0}\;{\hat{v}}_{1}\;{\hat{v}}_{2}\;\cdots \;{\hat{v}}_{N_{frame}-1}\right]$, which corresponds to the temporal basis functions rejected by the SVD filter as mentioned in our previous study [40]. Briefly, the main temporal frequency ${\bar{\omega }}_{SVD}$ was estimated as

(5) $${\bar{\omega }}_{SVD}=\frac{\sum_{\omega }\omega \left|\hat{R}\left(\omega \right)\right|}{\sum_{\omega }\left|\hat{R}\left(\omega \right)\right|},$$

where $\hat{R}\left(\omega \right)$ is the frequency spectrum of $\hat{r}\left(t\right)$ along the temporal direction t. The vector $\hat{r}$ of the rejected components is obtained as

(6) $$\hat{r}=\sum_{n=0}^{N_{low-rank}}\sigma_{n}{\hat{v}}_{n},$$

where $\sigma_{n}$ is the *n*-th singular value. While the components of the vector $\hat{r}$ are temporal samples of the rejected components, they are redefined as $\hat{r}\equiv \hat{r}\left(t\right)$.

**Figure 10:**
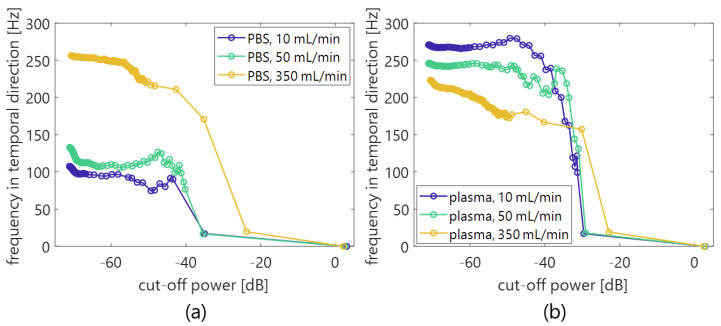
Mean temporal frequency in each cut-off power of singular value. (**a**,**b**) PBS and plasma samples.

**Figure 11:**
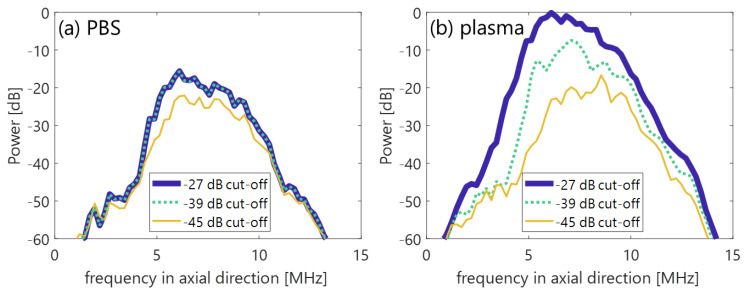
Power spectra in the axial direction. (**a**,**b**) PBS and plasma samples in the different low rank singular values at a selected flow rate of 10 mL/min. The spectra were computed in the typical frame at the center of the lumen.

**Figure 12:**
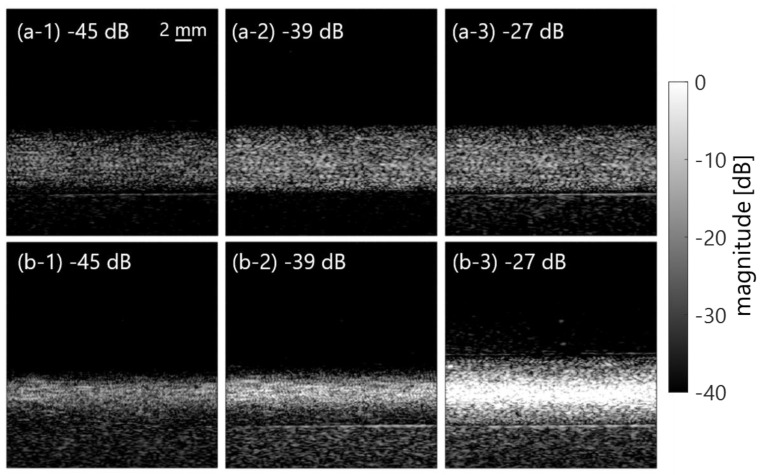
Typical B-mode images of PBS (**a**) and plasma (**b**) samples in the different clutter filter conditions at the flow rate of 10 mL/min.

**Figure 13:**
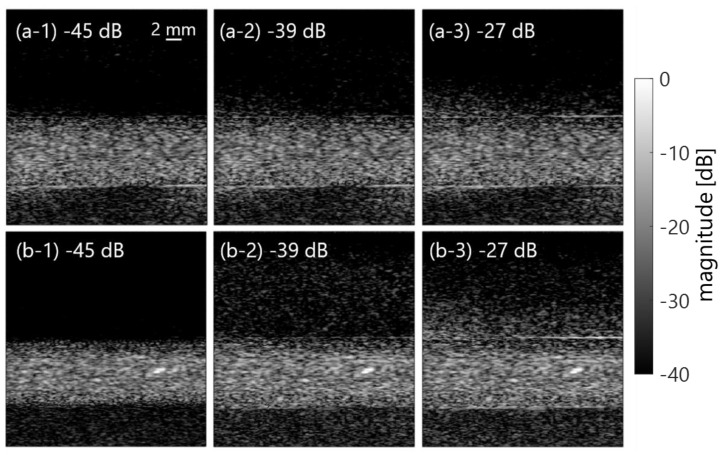
The same as in Figure 12, except at the flow rate of 350 mL/min.

**Figure 14:**
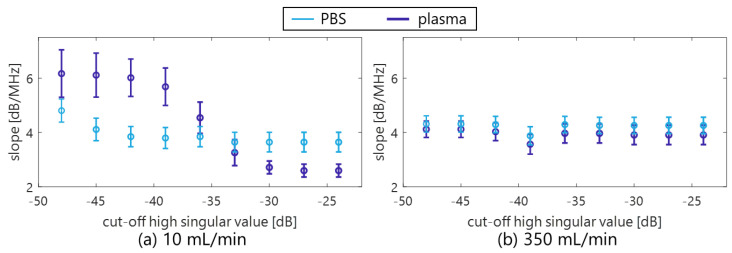
Effect of clutter filter conditions for the spectral slope in the porcine blood experiment at the flow rates of 10 mL/min (**a**) and 350 mL/min (**b**).

Figure 10 shows the mean frequency ${\bar{\omega }}_{SVD}$ in the porcine blood data, as is shown in Figure 2a. The components with low frequency under several 100 Hz (in Figure 10) were suppressed by the clutter filter at the cut-off value of approximately −25 to −35 dB (the second and the following plots from a plot at around 0 dB). Furthermore, low frequency components around 4 to 8 MHz (in the axial direction) dominantly decreased with decreasing cut-off values of the SVD filter, as are shown in the frequency spectra P(f) of Figure 11. For that reason, a high spectral slope and low MBF occurred in in vivo jugular vein in the constant phase of the shear rate. Such features were also confirmed in the porcine blood data, as are shown in Figure 12, Figure 13 and Figure 14. Typical B-mode images at selected flow rates of 10 and 350 mL/min are visualized in Figure 12 and Figure 13. Some different cut-offs of high singular values were selected in reference to Figure 2. At the flow rate of 350 mL/min (mean velocity and shear rate around 12 cm/s and 100 s^−1^), the clutter filter presumably distinguished between blood flow and tissue signals. Hence, the spectral slope was constant around 4 dB/MHz, independent of the cut-off property of singular values, as is shown in Figure 14b. However, the change in echogenicity occurred at the flow rate of 10 mL/min (mean velocity and shear rate around 1 cm/s and 10 s^−1^) for the different singular cut-off values. Additionally, the spectral slopes of the PBS and plasma samples increased from 2.2 to 6.0 dB/MHz with decreasing singular cut-off values in Figure 14a. Hence, the limitation of the condition of the clutter filter should be calibrated if the level of clutter rejection is uncertain. The criteria based on mean velocity, shear rate, and contrast might be necessary to reject the evaluation frame affected by the clutter filter. Another scenario is to measure with more wide-band *BSC* toward high frequency. Although the relationship between frequency range and penetration depth for this vascular imaging must be proven, high frequency ultrasound should emphasize backscattering power from the RBCs.

While the plasma sample in the low shear case presented the changes in the spectral slope and MBF in Figure 6, the physiological states were not confirmed in healthy subjects in in vivo situations. Diabetes [2], coronary artery disease [41], thrombosis [42], and hyperlipidemia [43] are associated with abnormally high levels of RBC aggregation. Chronic lower shear rates and flow disturbance in aging patients with diabetes might be distinct from the change in the hemorheological property, such as for the RBC aggregation and thrombus [44,45]. The RBC aggregation has been successively measured using line-by-line focused imaging in vivo in diabetic patients [46]. One of the potentials of high-frame-rate backscatter coefficient analysis for clinical applications will be conducted on a non-invasive quantitative analysis for anticoagulant therapy for diabetes subjects in future works.

## 5. Conclusions

The effect of a clutter filter in high-frame-rate ultrasonic *BSC* analysis was confirmed in the porcine blood and in in vivo jugular vein measurements. For the porcine blood experiment, the characteristics of the RBCs, such as aggregation and disaggregation, were evaluated as the change in the spectral slope and MBF of the *BSC* through the surrounding tissues with different clutter levels. The feasibility and reproducibility of this analysis were also compared in in vivo jugular veins in healthy subjects when the tissue and blood flow signals could be separated. High-frame-rate imaging with the clutter filter has the potential to characterize blood by means of the *BSC* analysis. In future works, the criteria based on flow features and image quality, such as the contrast, will provide feedback regarding the condition of the clutter filter to develop a robust analysis of the *BSC*.

## Data Availability

Not applicable.
